# Supplementary material for: IL-23 signaling prevents ferroptosis-driven renal immunopathology during candidiasis
Source: Nat Commun. 2022 Sep 22;13:5545. doi: 10.1038/s41467-022-33327-4 (PMC9500047; doi:10.1038/s41467-022-33327-4)
Supplement: Supplementary file 1 — Supplementary Information [file 41467_2022_33327_MOESM1_ESM.pdf]

Supplemental Figures

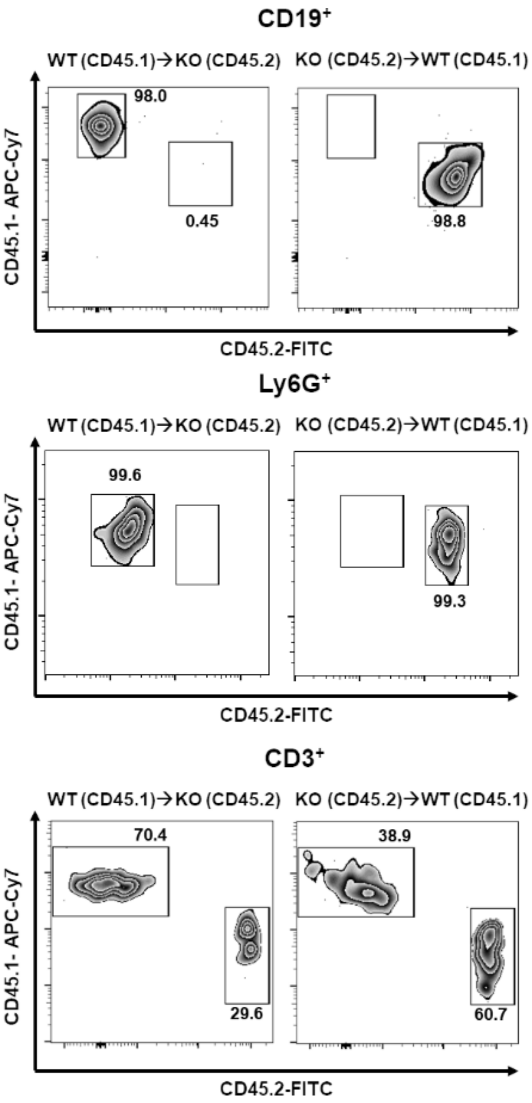

Fig. S1. Engraftment of bone marrow radiation chimeric mice before *C. albicans* infection. Whole bone marrow cells from WT (CD45.1; and CD45.2) and *Epha2*$^{-/-}$ (CD45.2) congenic mice were transferred to irradiated recipients and evaluated for engraftment 10 weeks later in blood samples. Each column of FACS plots corresponds to the subpopulation labeled at the top, analyzed for CD45.1 and CD45.2 expression. Data are representative of 7 mice.

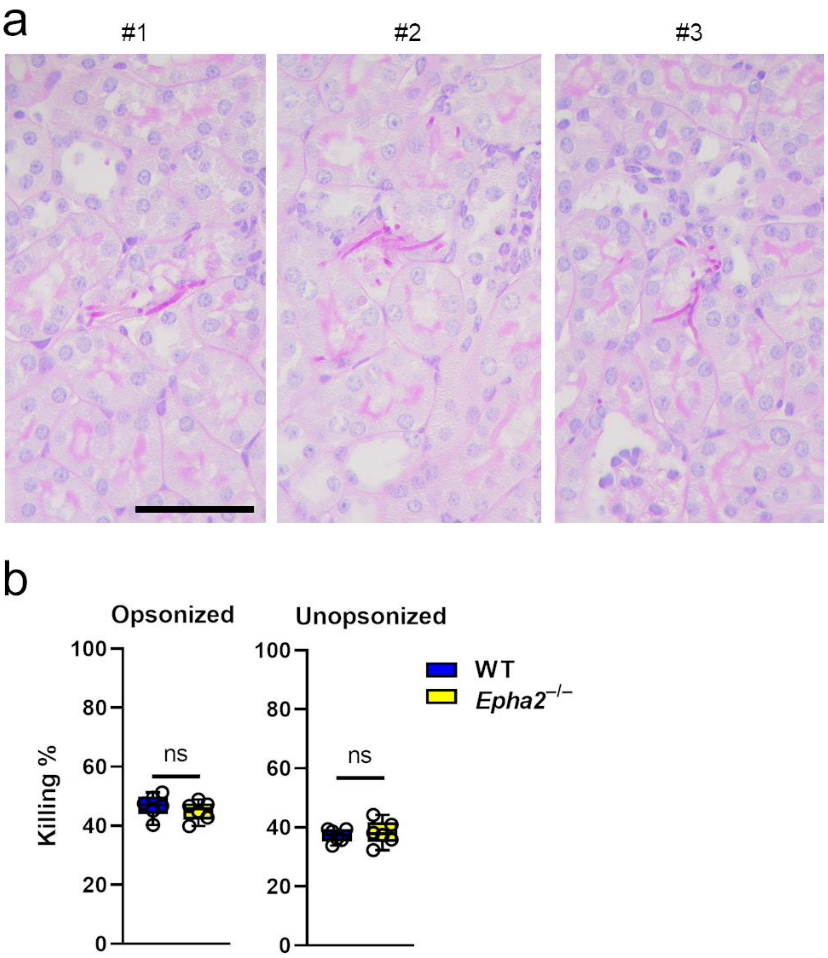

Fig. S2. a Morphology of *C. albicans* (SC5314) in kidneys 12 hours post infection. Shown are 3 PAS stained kidney sections (WT mice) from 3 different experiments. Scale bar 50 µm. **b** BM-neutrophil- mediated (opsonized and unopsonized) *C. albicans* killing. BM-neutrophils were isolated from WT and *Epha2*$^{-/-}$ mice. The percentage of organisms killed was determined by colony counting. N=3, triplicate. ns, not significant; Two-tailed Mann-Whitney test.

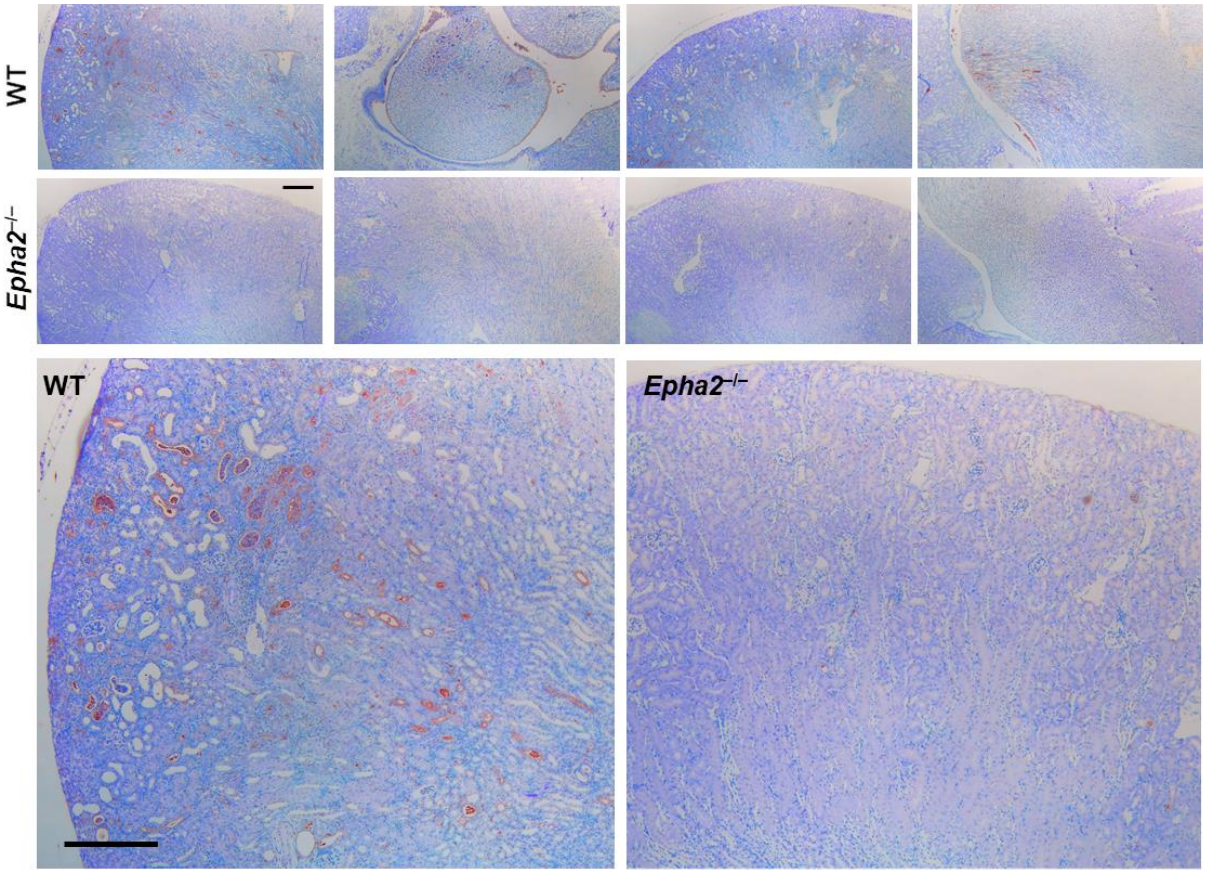

Fig. S3. Cell apoptosis in kidneys of WT and *Epha2*$^{-/-}$ mice during disseminated candidiasis. Apoptotic areas (TUNEL staining) are shown in brown. Pictures from two independent experiments. N= 6, combined data.

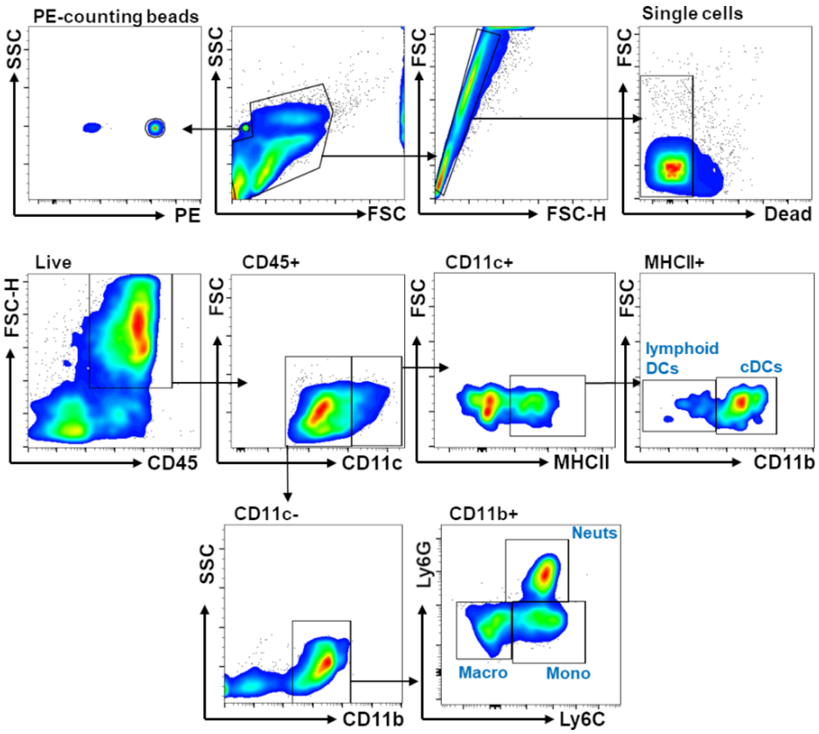

Fig. S4. Gating strategy used to quantify infiltrating immune cell in kidneys during disseminated candidiasis.

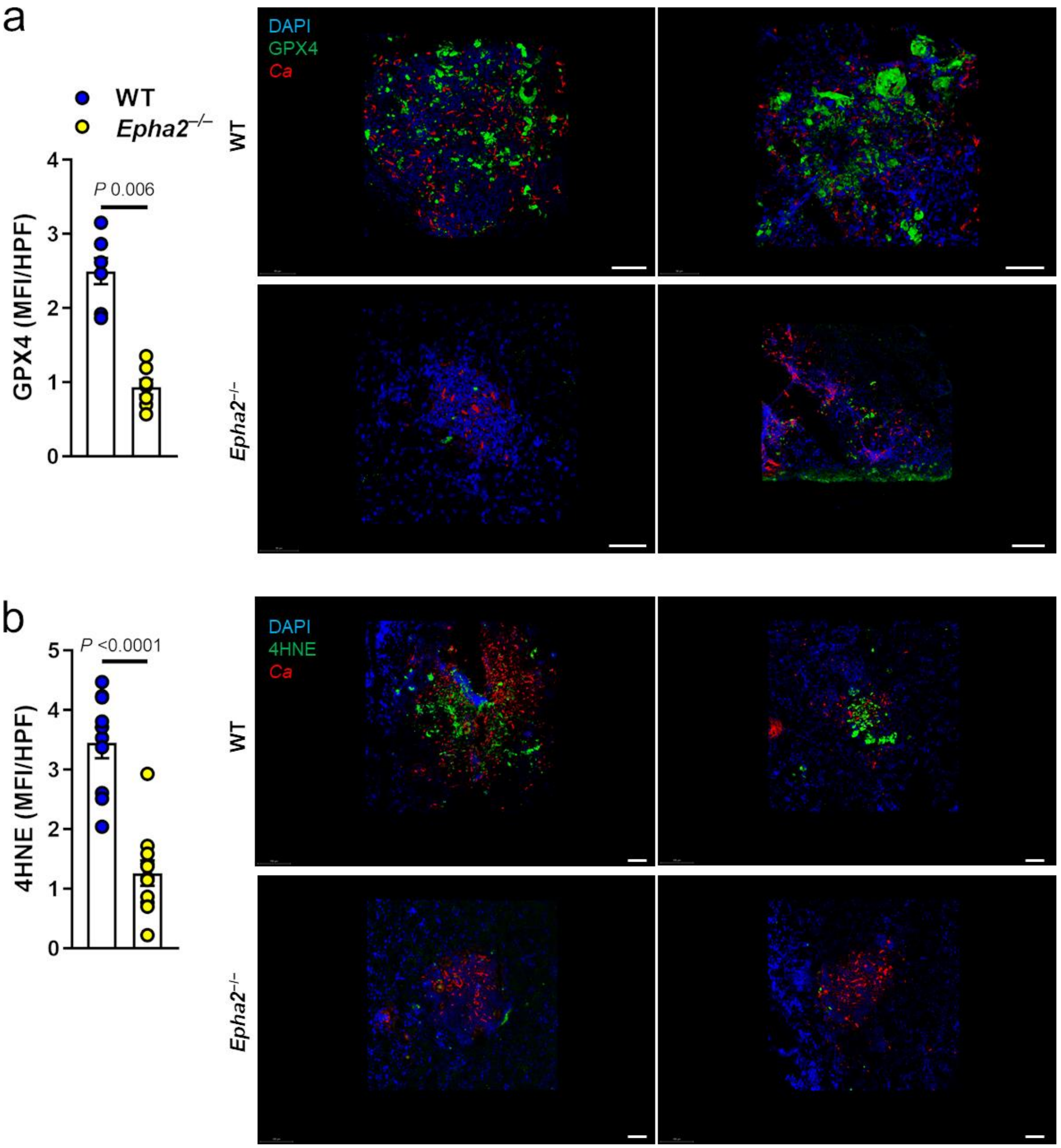

Fig. S5. GPX4 and 4HNE levels in infected kidneys of Wt and *Epha2*$^{-/-}$ mice. **a** (Left) GPX4 quantification of mean fluorescence intensity (MFI) per high power field (HPF). N=7 independent immunofluorescence images. Two-tailed Mann-Whitney Test. Mean ± SEM. (Right) GPX4 shown in green, *C. albicans* (*Ca*) in red. Tissue is visualized using DAPI. **b** 4HNE quantification of mean fluorescence intensity (MFI) per high power field (HPF). N=10 for WT and N=11 for *Epha2*$^{-/-}$ independent immunofluorescence images. Two-tailed Mann-Whitney Test. Mean ± SEM. (Right) 4HNE shown in green, *C. albicans* (*Ca*) in red. Tissue is visualized using DAPI. Scale bar 50 µm.

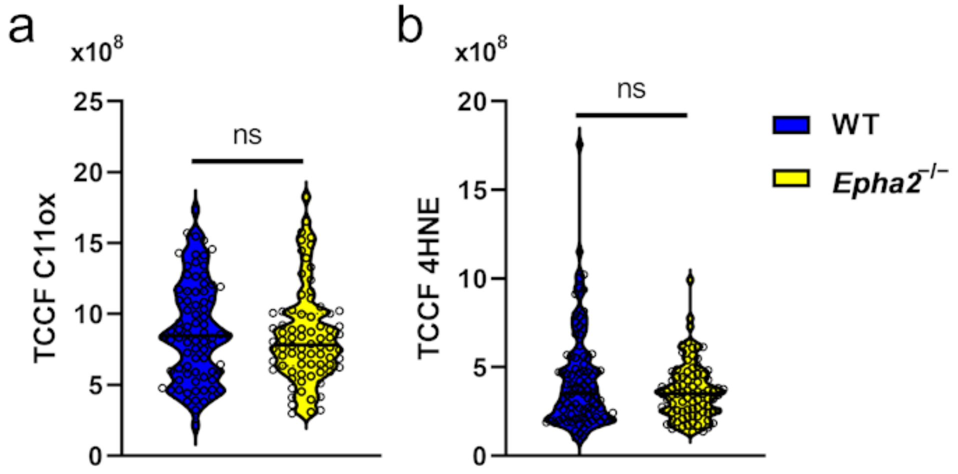

Fig. S6. BM-derived macrophages were infected with *C. albicans* (MOI 1) for 4 hours. Total C11ox fluorescence was quantified. Individual cells Quantification of total C11ox (**a**) and 4HNE (**b**) fluorescence. Individual cells in **a** N=79 for WT and N=78 for *Epha2*$^{-/-}$ and in **b** N=90 for WT and N=85 for *Epha2*$^{-/-}$. (N=78-90; 3 independent experiments) were identified and total fluorescence was measured using ImageJ. Ns, not significant. Two-tailed Mann-Whitney Test. Violin plot with median and quartiles.

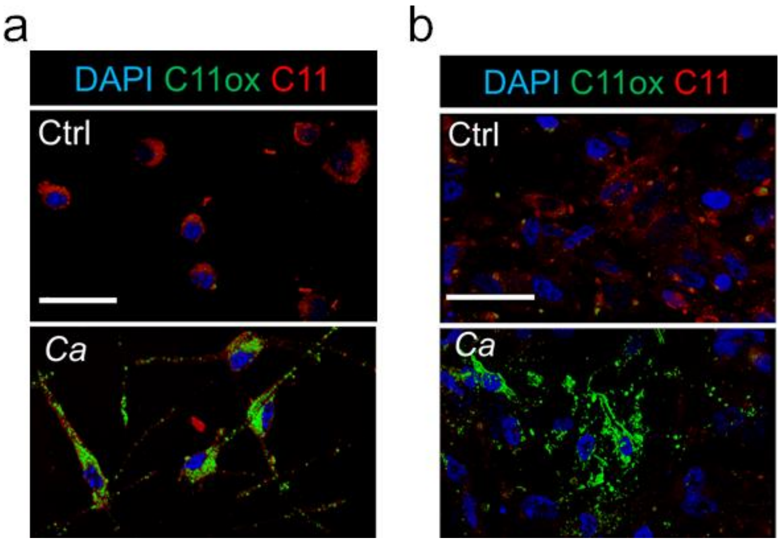

Fig. S7. BM-derived macrophages and renal tubular epithelial cells (RTECs) undergo ferroptosis during *Candida* infection. **a-b** Representative images of C11 oxidation of BM-derived macrophages (**a**) and renal tubular epithelial damage (**b**) during *C. albicans* infection. Host cells were infected with an MOI of 1 (BMDMs, 4 hours; RTECs, 2 hours). Scale bar 10 μm. N=3 independent experiments.

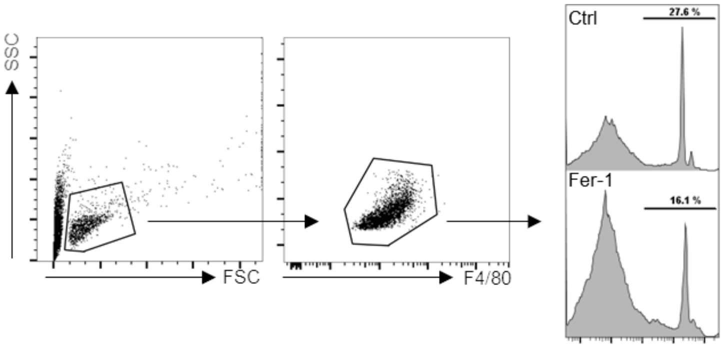

Fig. S8. Gating for macrophage death using F4/80 and propidium iodide. BMDMs were treated with Fer-1 and infected with *C. albicans* for 4 hours (MOI 5).

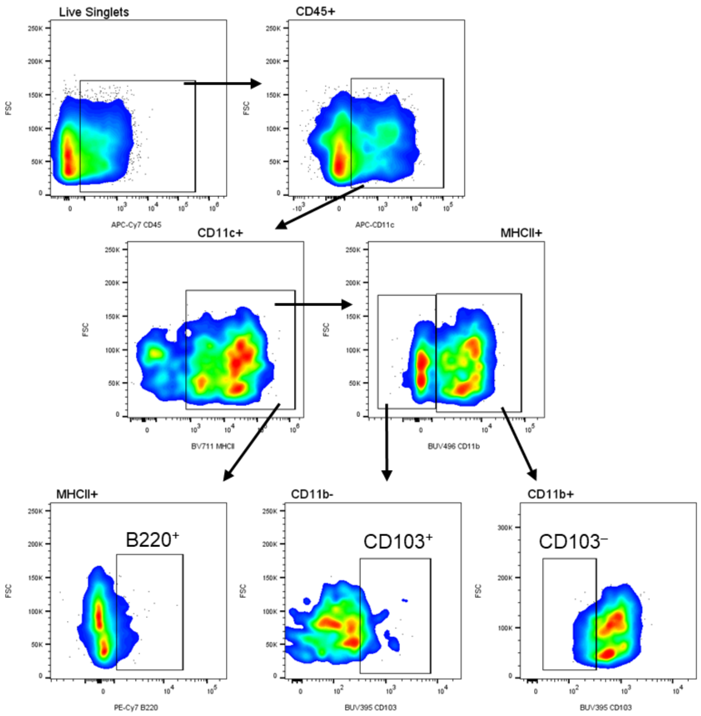

Fig. S9. Gating strategy used to quantify infiltrating DC populations in kidneys during disseminated candidiasis.

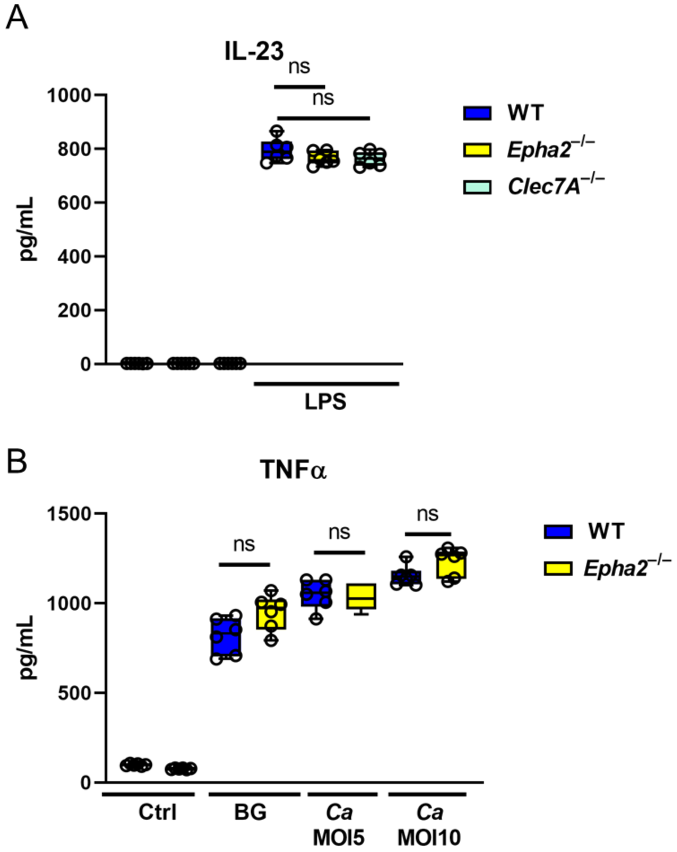

Fig. S10. Effect of DCs Epha2 deficiency on IL-23 and TNFα secretion. BMDCs were stimulated with LPS, β-glucan (BG), or heat killed Candida (*Ca*) for 24 hours. **A** IL-23 and **B** TNFα levels in supernatants were determined with ELISA. N=3, duplicate. Ns, not significant; Two-tailed Mann-Whitney Test. Box-and-whisker plots indicating median, 25th/75th percentiles, and the minimum/maximum values.

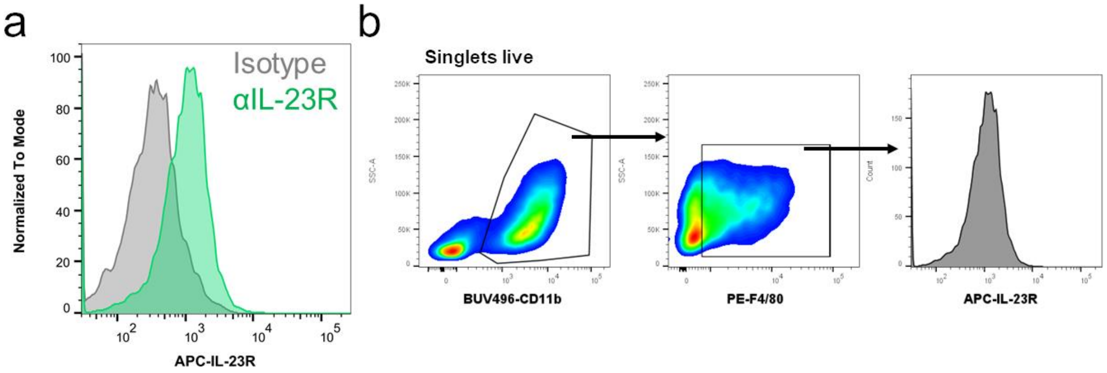

Fig. S11. CD11b$^+$ F4/80$^+$ macrophages express the IL-23 receptor during infection. WT mice were infected with 2.5 x10^5 *Candida* yeast cells. After 3 days of infection. Single cell suspensions of kidneys were stained for IL-23R expression. **a** Histogram shows representative IL-23R surface expression of live single CD11b$^+$ F4/80$^+$ macrophages Histogram of IL-23R surface expression. **b** Gating strategy used to determine IL-23R expression in CD11b$^+$ F4/80$^+$ macrophages in kidneys during disseminated candidiasis.

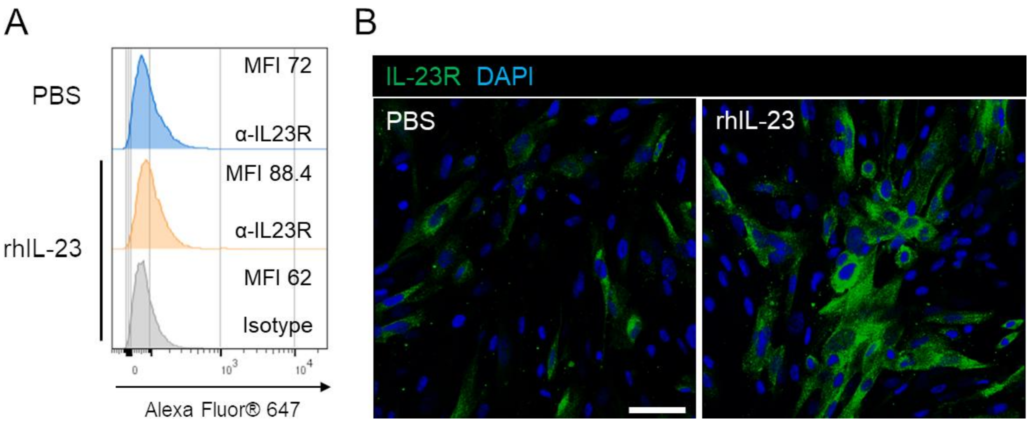

Fig. S12. IL-23R expression in RTECs after IL-23 treatment. **A** Histogram plots of IL-23R surface expression treatment with rhIL-23 (250 ng/ml) for 48 hours. Median fluorescence intensity (MFI) is indicated in plots. **B** Representative images of IL-23R expression of RTECs treated with rhIL-23 (250 ng/ml) for 18 hours. Scale bar 50 μm. Two independent experiments.

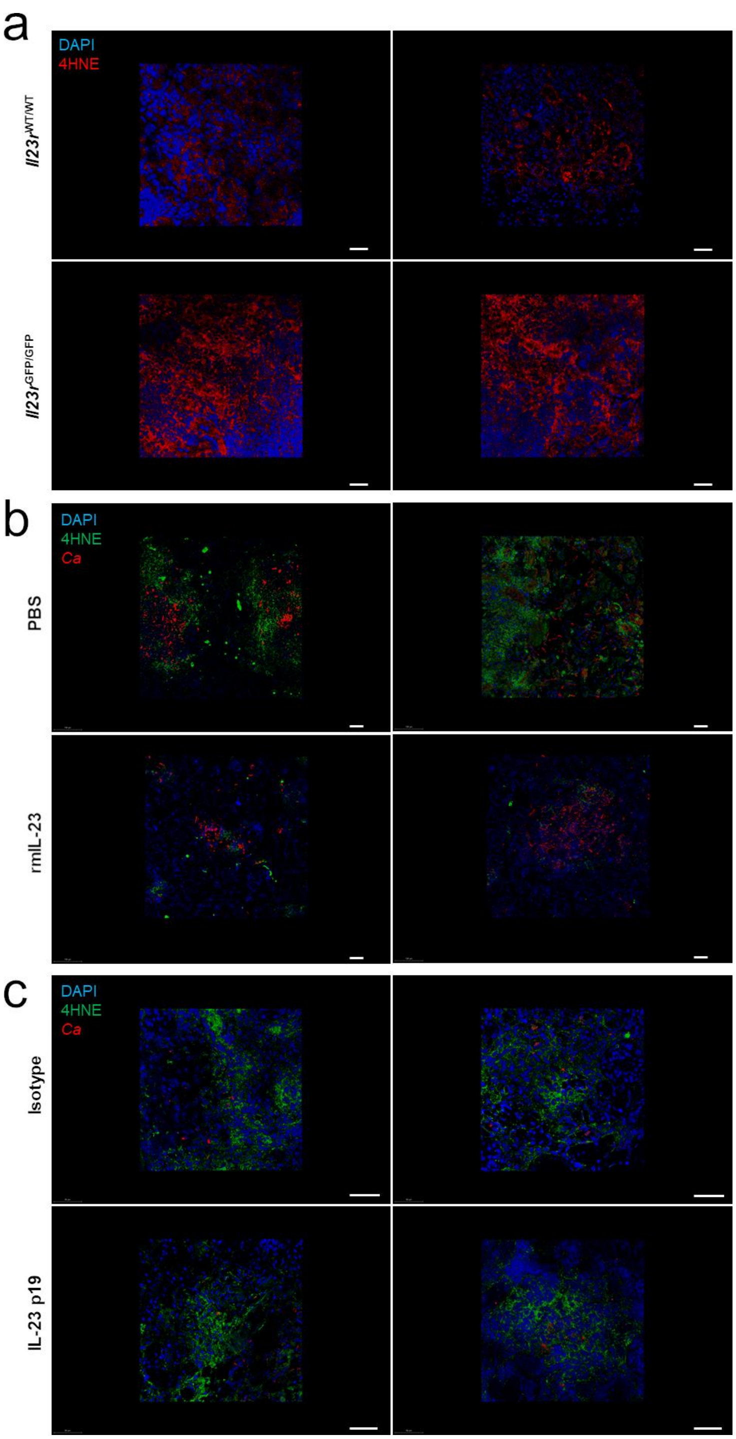

Fig. S13. 4HNE in infected kidneys of indicated mice. **A** Lipid peroxidation (4HNE) in infected kidneys of *Il23r*$^{WT/WT}$ and *Il23r*$^{GFP/GFP}$ mice after 3 days of infection. 4HNE shown in red, tissue stained with DAPI (blue). Scale bar 100 µm. **B** Lipid peroxidation in infected kidneys after 3 days of infection using 4HNE. Single rmIL-23 treatment at day 2 relative to infection. 4HNE shown in green, *C. albicans* (*Ca*) in red. Tissue is visualized using DAPI. Scale bar 100 µm. **C** IL-23 p19 depletion *Epha2*$^{-/-}$ mice. Lipid peroxidation in infected kidneys after 3 days of infection using 4HNE. 4HNE shown in green, *C. albicans* (Ca) in red. Tissue is visualized using DAPI. Scale bar 50 µm.

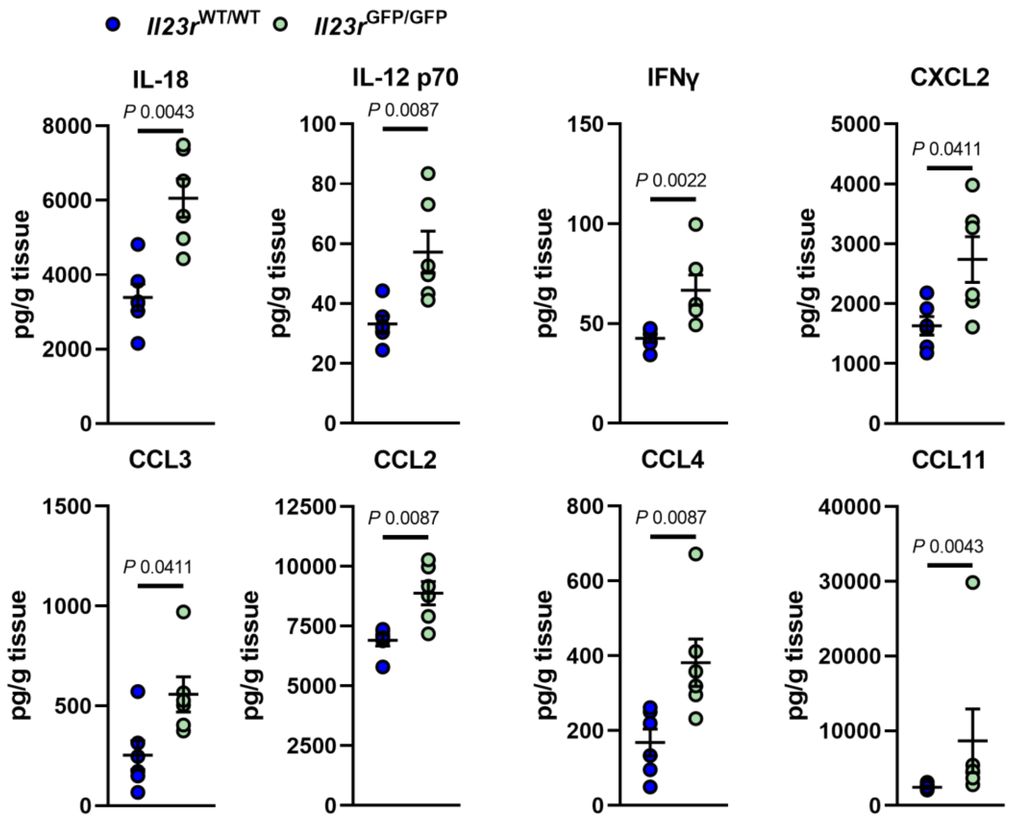

Fig. S14. Levels of indicated cytokines in infected kidneys of *Il23r*$^{WT/WT}$ and *Il23r*$^{GFP/GFP}$ mice after 3 days of infection. N=6; combined data of two independent experiments. Two-tailed Mann-Whitney Test. Dot blot shows mean ± SEM.

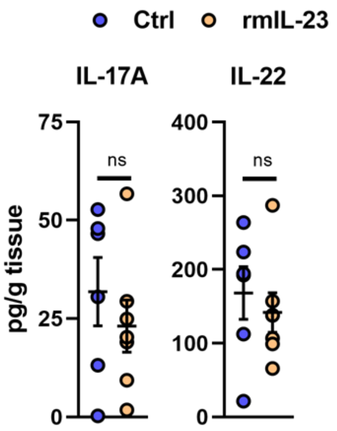

Fig. S15. Renal IL-17A and IL-22 levels in mice treated with recombinant murine IL-23. Single rmIL-23 treatment at day 2 post infection. Kidney harvested at day 3 post infection. N=6 for vehicle-treated mice and N=7 for rmIL-23-teated mice; combined data of two independent experiments. Mice were treated with recombinant murine IL-23 (rmIL-23) or vehicle (Ctrl; PBS). Two-tailed Mann-Whitney Test. Bar graph shows mean ± SEM.

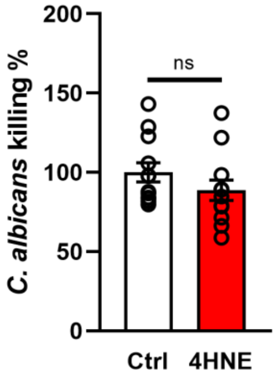

Fig. S16. 4HNE has no effect on *C. albicans*. C. albicans cells were incubated with 50 μm 4HNE for 6 hours. N=12 independent experiments, combined data. Ns, not significant; Two-tailed Mann-Whitney Test. Bar graph shows mean ± SEM.

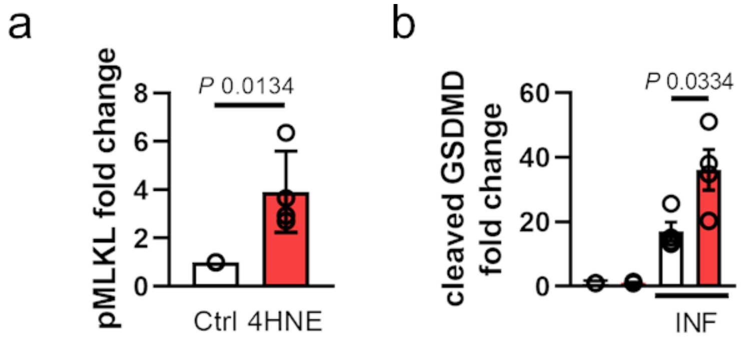

Fig. S17. Densitometric analysis of proteins shown in Fig. 10. **a** MLKL phosphorylation in BMDMs treated with 4HNE. **b** Cleaved Gasdermin-D relative to full length protein. BMDM were infected with C. albicans yeast for 2 hours followed by incubation with 4HNE. Data are the mean ± SD of 4 independent immunoblots. Unpaired t test.

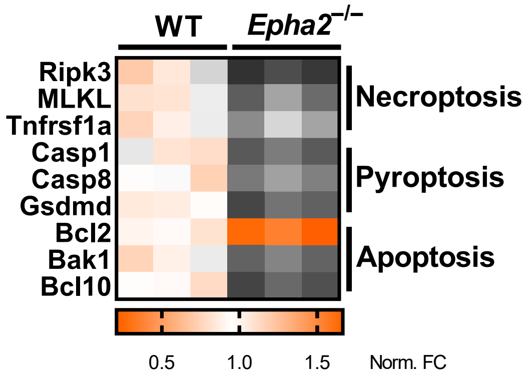

Fig. S18. Heatmap of key genes involved in necroptosis, pyroptosis, and apoptosis. Shown are normalized fold changes (FC). RNASeq was performed on mRNA isolated from kidneys of WT and *Epha2*$^{-/-}$ mice after 3 days of infection. N=3 per mouse strain.

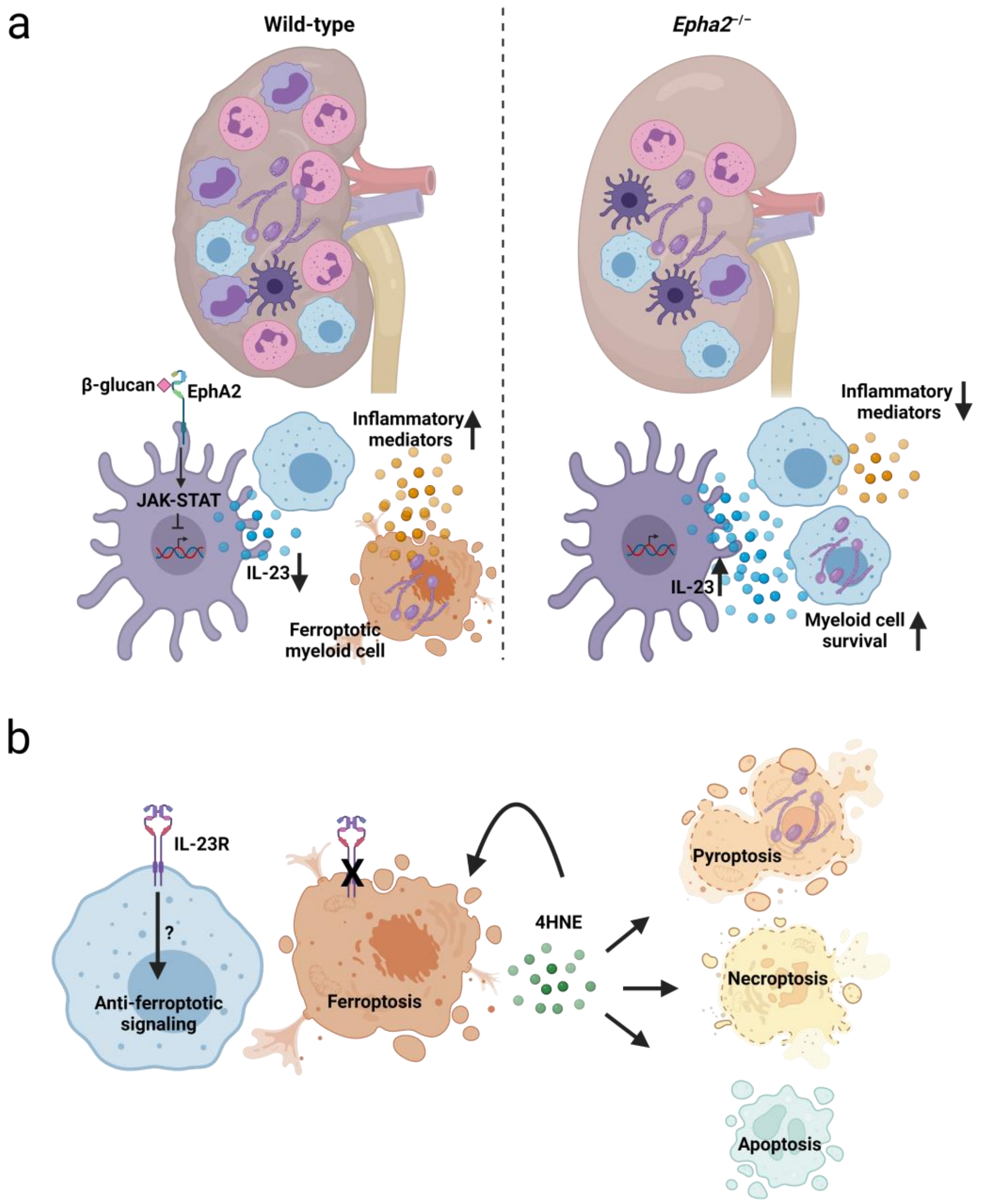

Fig. S19. Graphical summary. **a** During disseminated candidiasis, EphA2 in dendritic cells induces JAK-STAT signaling to limit IL-23 secretion and consequently promotes inflammation via ferroptotic cell death. **b** IL-23 receptor signaling prevents ferroptotic cell death via an unknown mechanism. 4HNE released by ferroptotic macrophages induces apoptosis, ferroptosis, necroptosis, and pyroptosis in adjacent cells. Created with BioRender.com.

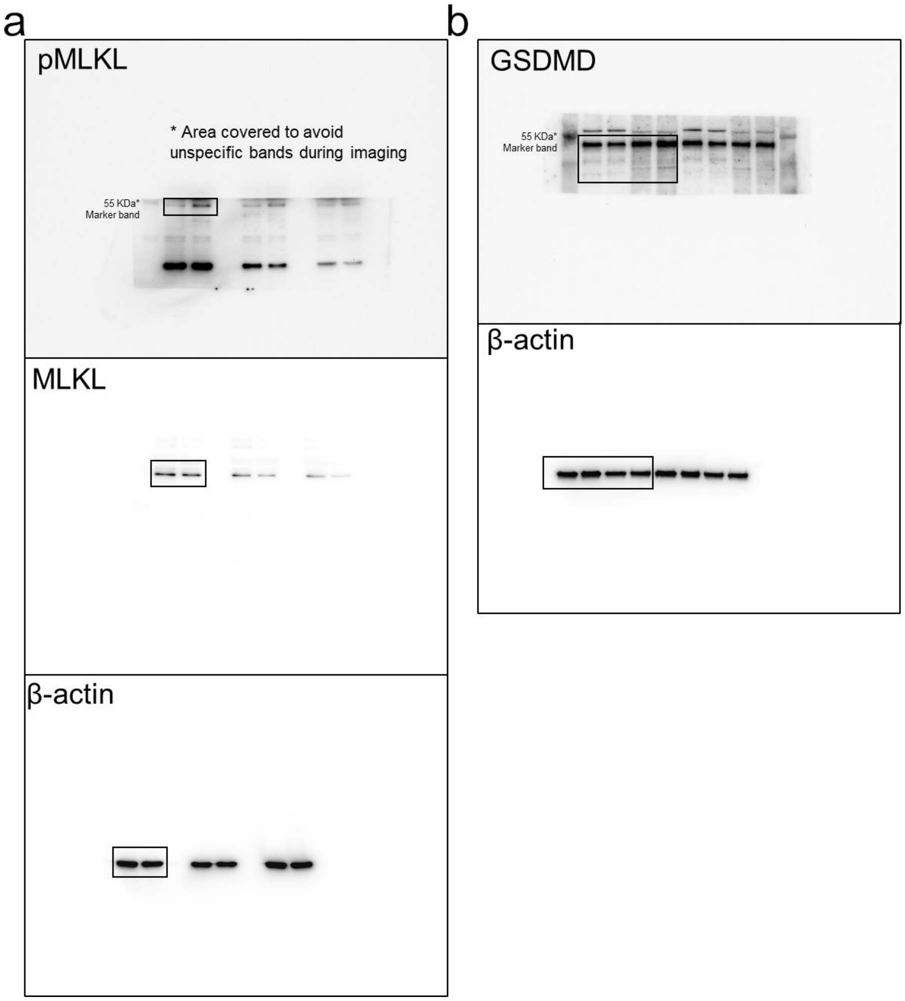

Fig. S20. Uncropped raw immunoblots shown in Fig.10. Indicated boxes show the cropped immunoblots in figure 10.
