# Supplementary material for: IL-23 signaling prevents ferroptosis-driven renal immunopathology during candidiasis
Source: Nat Commun. 2022 Sep 22;13:5545. doi: 10.1038/s41467-022-33327-4 (PMC9500047; doi:10.1038/s41467-022-33327-4)
Supplement: Supplementary file 3 — Reporting Summary [file 41467_2022_33327_MOESM3_ESM.pdf]

nature portfolio

Corresponding author(s): Marc Swidergall

Last updated by author(s): Aug 25, 2022

Reporting Summary

Nature Portfolio wishes to improve the reproducibility of the work that we publish. This form provides structure for consistency and transparency in reporting. For further information on Nature Portfolio policies, see our Editorial Policies and the Editorial Policy Checklist.

Statistics

For all statistical analyses, confirm that the following items are present in the figure legend, table legend, main text, or Methods section.

n/a	Confirmed	
☐	☒	The exact sample size (*n*) for each experimental group/condition, given as a discrete number and unit of measurement
☐	☒	A statement on whether measurements were taken from distinct samples or whether the same sample was measured repeatedly
☐	☒	The statistical test(s) used AND whether they are one- or two-sided<br>*Only common tests should be described solely by name; describe more complex techniques in the Methods section.*
☒	☐	A description of all covariates tested
☐	☒	A description of any assumptions or corrections, such as tests of normality and adjustment for multiple comparisons
☐	☒	A full description of the statistical parameters including central tendency (e.g. means) or other basic estimates (e.g. regression coefficient) AND variation (e.g. standard deviation) or associated estimates of uncertainty (e.g. confidence intervals)
☐	☒	For null hypothesis testing, the test statistic (e.g. F, t, r) with confidence intervals, effect sizes, degrees of freedom and P value noted<br>*Give P values as exact values whenever suitable.*
☒	☐	For Bayesian analysis, information on the choice of priors and Markov chain Monte Carlo settings
☒	☐	For hierarchical and complex designs, identification of the appropriate level for tests and full reporting of outcomes
☐	☒	Estimates of effect sizes (e.g. Cohen's *d*, Pearson's *r*), indicating how they were calculated

Our web collection on statistics for biologists contains articles on many of the points above.

Software and code

Policy information about availability of computer code

Data collection	The following standard software provided by instrument suppliers was used for data collection:<br><br>Histology: PROGRES GRYPHAX Software version 1.1.8.153<br>Flow cytometry: BD FACSDiva Software V.8.0<br>Cytokine bead array: Luminex Xpotent Software V4.2<br>Plate reader: Gen5 version 3.11<br>Confocal: LAS X Leica Application Suite X v 3.5.2.18963
Data analysis	GraphPad Prism (Version 9.4.0)<br>FlowJo (Version 10.8.1)<br>ImageJ (V1.8)

For manuscripts utilizing custom algorithms or software that are central to the research but not yet described in published literature, software must be made available to editors and reviewers. We strongly encourage code deposition in a community repository (e.g. GitHub). See the Nature Portfolio guidelines for submitting code & software for further information.

Data

Policy information about availability of data

All manuscripts must include a data availability statement. This statement should provide the following information, where applicable:

- Accession codes, unique identifiers, or web links for publicly available datasets
- A description of any restrictions on data availability
- For clinical datasets or third party data, please ensure that the statement adheres to our policy

The authors declare that the data supporting the findings of this study are available within the paper, the accompanying supplementary information files, and the source data (Source Data file). The high-throughput sequencing data from this study have been deposited with links to BioProject accession number PRJNA773053 and PRJNA773073 in the NCBI BioProject database. The reference genome of Mus musculus (GRCm38/mm10) and gene model annotation files were downloaded from NCBI/UCSC/Ensembl.

Field-specific reporting

Please select the one below that is the best fit for your research. If you are not sure, read the appropriate sections before making your selection.

☒ Life sciences ☐ Behavioural & social sciences ☐ Ecological, evolutionary & environmental sciences

For a reference copy of the document with all sections, see nature.com/documents/nr-reporting-summary-flat.pdf

Life sciences study design

All studies must disclose on these points even when the disclosure is negative.

Sample size	For in vivo experiments (fungal burden, immune cell recruitment, cytokines) we will use 6 mice (Knockouts or treatment) and 6 controls. In previous studies the response within each subject group was normally distributed with standard deviation 0.25. If the true difference in the experimental and control means is 0.5 (e.g., 50% increase or decrease), we will be able to reject the null hypothesis that the population means of the experimental and control groups are equal with probability (power) >0.85. The Type I error probability associated with this test of this null hypothesis is 0.05. All experiments used power analysis to determine the appropriate number of mice.<br>At least three biological replicates were performed for all in vitro experiments unless otherwise indicated. Data were compared by Mann-Whitney corrected for multiple comparisons using GraphPad Prism (v. 9) software. P values < 0.05 were considered statistically significant.
Data exclusions	No data were excluded
Replication	All experimental findings were reproduced (in vivo: at least two times; in vitro at least three times)
Randomization	Fer-1 treatment and IL-23 p19 depletion: The mice were randomized to the two different treatment groups (drug or antibody vs. vehicle).
Blinding	Researchers were not blinded to the experimental groups because the endpoints (survival, fungal burden, cytokine levels, etc.) were objective measures of disease severity. However, the outcomes were quantitative and not subjective.

Reporting for specific materials, systems and methods

We require information from authors about some types of materials, experimental systems and methods used in many studies. Here, indicate whether each material, system or method listed is relevant to your study. If you are not sure if a list item applies to your research, read the appropriate section before selecting a response.

Materials & experimental systems

n/a	Involved in the study	
☐	☒	Antibodies
☐	☒	Eukaryotic cell lines
☒	☐	Palaeontology and archaeology
☐	☒	Animals and other organisms
☒	☐	Human research participants
☒	☐	Clinical data
☒	☐	Dual use research of concern

Methods

n/a	Involved in the study	
☒	☐	ChIP-seq
☐	☒	Flow cytometry
☒	☐	MRI-based neuroimaging

Antibodies

Antibodies used	CD45 (30-F11, AB_398672BD, Biosciences Cat. No. 559864, Lot: 8277680); Dilution: 1:100<br>Ly6C (AL-21, AB_394628, BD Biosciences Cat. No. 553104, Lot: 7324924); Dilution: 1:100

Ly6C (HK1.4, AB_2562177, BioLegend Cat. No. 128031, Lot: B284703)
Ly6G (1A8, AB_1186104, BioLegend Cat. No. 127607, Lot: B268001; AB_1186104, BioLegend Cat. No. 127607, Lot: B277149); Dilution: 1:100
CD11b (M1/70; AB_312790, BioLegend Cat. No. 101207, Lot: B269029; AB_312789, BioLegend Cat. No. 101206, Lot: B206637; AB_2874105, BD Biosciences Cat. No. 749864, Lot: 2084552); Dilution: 1:100
CD11c (N418, AB_313779, BioLegend Cat. No. 117310, Lot: B280313); Dilution: 1:100
MHCII (M5/114.15.2, AB_2565976, BioLegend Cat. No. 107643, Lot: B299330); Dilution: 1:100
CD206 (C068C2, AB_10900988 (BioLegend Cat. No. 141703, Lot: B246216); Dilution: 1:100
B220 (RA3-6B2, AB_313004, BioLegend Cat. No. 103221, Lot: B270361); Dilution: 1:100
CD103 (2E7, AB_2872683, BD Biosciences Cat. No. 748253, Lot: 2084551); Dilution: 1:100
mIL-23R (Clone # 753317, R&D Systems, Fab16861, Lot: ACYY0322031); Dilution: 1:100
mIL-23R (Clone # 320244, R&D Systems, IC18871P, Lot: ABSQ0321091); Dilution: 1:100
F4/80 (BM8, AB_893486, BioLegend Cat. No. 123110, Lot: B357448); Dilution: 1:100
anti-IL-23R antibody (EPR22838-4, Abcam Cat. No. ab222104, Lot: GR3281510-1); Dilution: 1:100
Alexa Fluor 647 Donkey anti-rabbit IgG (Poy4064, AB_2563202, BioLegend Cat. No. 406414, Lot: B299256); Dilution: 1:100
anti-pMLKL (S345) (D6E3G, Cell signaling Cat. No. 37333, Lot: 2); Dilution: 1:1000
anti-MLKL (D6W1K, Cell signaling Cat. No. 37705, Lot:4); Dilution: 1:1000
anti-cleaved N-terminal GSDMD antibody (EPR20829-408, Abcam Cat. No. ab215203, Lot: GR3360943-5); Dilution: 1:1000
anti-rabbit IgG HRP (Cell Signaling Cat. No. 7074, Lot: 30); Dilution: 1:10000
anti-4 Hydroxynonenal antibody (Abcam Cat# ab46545, RRID:AB_722490): Dilution 1:100
anti-Glutathione Peroxidase 4 antibody [EPNCIR144] (Abcam Cat# ab125066, RRID:AB_10973901); Dilution 1:100

Validation

All antibodies were purchased from commercial sources. BD and BioLegend antibodies are tested by ELISA capture. Cell Signaling Technology Abs are tested for functionality, specificity, and sensitivity using siRNA and heterozygous knockout assays, mass spectrometry and in situ hybridization, IP, ChIP, and ChIP-seq, ELISA, peptide dot blots, peptide blocking, and protein arrays.

Eukaryotic cell lines

Policy information about cell lines

Cell line source(s)	Primary human renal tubular epithelial cells (ATCC)
Authentication	Pan-Cytokeratin (+), y-glutamyltransferase-1 (GGT-1) (+), TE-7 (-) (verifed by ATCC)
Mycoplasma contamination	Tested and found to be uncontaminated.
Commonly misidentified lines (See ICLAC register)	n/a

Animals and other organisms

Policy information about studies involving animals; ARRIVE guidelines recommended for reporting animal research

Laboratory animals

-Epha2–/– (B6-Epha2tm1Jrui/J; CD45.2) mice were provided by A. Wayne Orr
-C57BL/6 control mice (wild type; CD45.2) and B6.SJL-Ptprca Pepcb/BoyJ (WT; CD45.1) purchased from The Jackson laboratory
-Il23rWT/GFP purchased from The Jackson laboratory. Il23rWT/GFP were bred to Il23rWT/GFP to obtain Il23rWT/WT and Il23rGFP/GFP mice
-Clec7a–/– mice were purchased from The Jackson Laboratory

Wild animals

No wild animals were used in the study

Field-collected samples

No field collected samples were used in the study

Ethics oversight

All animal work was approved by the Institutional Animal Care and Use Committee (IACUC) of the Lundquist Institute at Harbor-UCLA Medical Center. Protocol numbers: #30927; #32524

Note that full information on the approval of the study protocol must also be provided in the manuscript.

Flow Cytometry

Plots

Confirm that:

☒ The axis labels state the marker and fluorochrome used (e.g. CD4-FITC).

☒ The axis scales are clearly visible. Include numbers along axes only for bottom left plot of group (a 'group' is an analysis of identical markers).

☒ All plots are contour plots with outliers or pseudocolor plots.

☒ A numerical value for number of cells or percentage (with statistics) is provided.

Methodology

Sample preparation	Mice were infected with C. albicans strain SC5314. After 3 days of infection, mice were anesthetized using ketamine/xylazine and perfused with 10 ml of PBS before kidney harvesting. Kidneys were finely minced and digested at 37°C in digestion solution (RPMI 1640 with 20 mM HEPES [Gibco] without serum) containing liberase TL (Roche) and grade II DNAse I (Roche) for 20 minutes with shaking. Digested tissue was passed through a 70-µm filter and washed. The remaining red blood cells were lysed with ACK lysis buffer (Lonza). The cells were suspended in 40% Percoll (GE Healthcare). The suspension was overlaid on 70% Percoll and centrifuged at 836 g for 30 minutes at room temperature. The leukocytes and nonhematopoietic cells at the interphase were isolated, washed 3 times in FACS buffer (0.5% BSA and 0.01% NaN3 in PBS). After washing with FACS buffer, the cell suspension was stained with a Fixable Viability Stain 510 (BD Biosciences), washed, and resuspended in FACS buffer. The single-cell suspensions were then incubated with rat anti-mouse CD16/32 (2.4G2; BD Biosciences) for 10 minutes (1:100) in FACS buffer at 4°C to block Fc receptors, washed and followed by surface antigen staining.
Instrument	BD FACSymphony™ A5 Cell Analyzer
Software	FACS Diva (BD Biosciences) and FlowJo software (Treestar)
Cell population abundance	For RNA sequencing of BMDCs, cells were purified using negative magnetic bead selection (MojoSort Mouse Pan Dendritic Cell Isolation Kit, BioLegend). Purity of DCs was verified by flow cytometry of CD11c positive cells > 95%
Gating strategy	The initial gate events were visualized using a FSC-A/SSC-A dot plot. Debris were excluded. Cell doublets were excluded using the FSC-A and FSC-W. Singlets were gated on Live/Dead. Leukocytes were identified as CD45+, and further divided into CD11c + (DCs) or negative cells. CD11c+ cells were further divided into MHCII positive cells, and divided into CD11b+ and CD11b- cells. CD11c- cells divided into CD11b+ cells und further divided by Ly6G and Ly6C to identify Ly6Chigh Ly6G– inflammatory monocytes and Ly6C+ Ly6G+ neutrophils, and Ly6c-Ly6G macrophages. In some experiments, CD11c+ cells were divided into MHCII + and MHCII- cells and further stained for CD103.

☒ Tick this box to confirm that a figure exemplifying the gating strategy is provided in the Supplementary Information.
